# Asexual and sexual reproduction are two separate developmental pathways in a *Termitomyces* species

**DOI:** 10.1098/rsbl.2020.0394

**Published:** 2020-08-12

**Authors:** Sabine M. E. Vreeburg, Norbert C. A. de Ruijter, Bas J. Zwaan, Rafael R. da Costa, Michael Poulsen, Duur K. Aanen

**Affiliations:** 1 Department of Plant Sciences, Laboratory of Genetics, Wageningen University, Wageningen, The Netherlands; 2 Department of Plant Sciences, Laboratory of Cell Biology, Wageningen University, Wageningen, The Netherlands; 3 Section for Ecology and Evolution, Department of Biology, University of Copenhagen, Copenhagen, Denmark

**Keywords:** *Termitomyces*, nodules, symbiosis, mushroom formation, mutualism, fungus-growing termites

## Abstract

Although mutualistic symbioses per definition are beneficial for interacting species, conflict may arise if partners reproduce independently. We address how this reproductive conflict is regulated in the obligate mutualistic symbiosis between fungus-growing termites and *Termitomyces* fungi. Even though the termites and their fungal symbiont disperse independently to establish new colonies, dispersal is correlated in time. The fungal symbiont typically forms mushrooms a few weeks after the colony has produced dispersing alates. It is thought that this timing is due to a trade-off between alate and worker production; alate production reduces resources available for worker production. As workers consume the fungus, reduced numbers of workers will allow mushrooms to ‘escape’ from the host colony. Here, we test a specific version of this hypothesis: the typical asexual structures found in all species of *Termitomyces*—nodules—are immature stages of mushrooms that are normally harvested by the termites at a primordial stage. We refute this hypothesis by showing that nodules and mushroom primordia are macro- and microscopically different structures and by showing that in the absence of workers, primordia do, and nodules do not grow out into mushrooms. It remains to be tested whether termite control of primordia formation or of primordia outgrowth mitigates the reproductive conflict.

## 1. Introduction

All known species of the basidiomycete genus *Termitomyces* grow in a remarkable, obligate symbiosis with termites of the subfamily Macrotermitinae [1]. This farming symbiosis, in which termite hosts grow fungal symbionts for food in exchange for substrate and shelter, has attracted the interest of many ecologists and evolutionary biologists (e.g. [2–6]). A major conundrum in the termite–fungus symbiosis is how the reproductive interests of host and symbiont are aligned, despite their independent dispersal in most fungus-growing termite species [1,7].

*Termitomyces* fungi have both an asexual and a sexual life cycle [3]. The asexual cycle is the dominant lifecycle in a colony, while the sexual life cycle is required for symbiont dispersal to new colonies [8]. Within a nest the fungus is grown on airy structures of plant substrate, called the fungus comb. The fungus colonizes the comb and subsequently forms spherical structures that contain asexual spores: nodules. These nodules are consumed by termites together with plant material and defaecated to form new fungus comb, thereby completing the asexual cycle [9]. For sexual reproduction, the fungus forms sexual fruiting bodies: mushrooms [10]. These mushrooms have their origin in the fungus comb and pierce their way up to the surface of the termite mound. Once matured, they spread sexual spores throughout the environment, which are picked up by foraging termites to inoculate newly founded, fungus-less termite colonies [3,11,12].

Paradoxically, while most fungus-growing termite species are dependent on acquiring their symbiont from spores in the environment [1,13], it is not in the short-term interest of any individual termite colony to allow its fungus to fruit [8]. Production of fruiting bodies wastes resources that could otherwise have been allocated to growth of the colony and ultimately to more alates. This has led multiple researchers to argue that the termites actively suppress fruiting body formation of their fungal symbiont [2,6,8,14]. Indeed it seems plausible that fewer workers can be produced to maintain the fungus comb, when alates are produced by a colony, and fewer mushroom initials will be eaten [5,6,14,15]. As a more specific corollary of this idea, it has been speculated that, in response to consumption of mushrooms at a primordial stage, the fungus would have evolved gut-resistant asexual spores on the unripe mushrooms, leading to the typical asexual structures found in all species of *Termitomyces*: nodules [6,9,10,14]. According to this hypothesis, these ubiquitous nodules are the initials of mushrooms that can develop into sexual fruiting bodies if not eaten by termites [13,16,17].

Here, we set out to test the latter assumption. Under the assumption that nodules are unripe mushrooms, nodules on fungus comb fragments incubated in the absence of termites should develop into mushrooms. Also, since the inner structure of initials of other basidiomycetes shows clear mushroom features at very early stages [18,19], we hypothesized that if the nodules were equivalent to these stages of mushroom formation, they should show similar differentiation into mushroom.

## 2. Material and methods

### (a) Excavations and fungus comb incubations

A minimum of 15 fungus comb samples were excavated from 25 mature *Macrotermes natalensis* colonies in January and February 2015, 2016 and 2018. We chose to study the combs of this particular termite species, because it has been found that all *Termitomyces* strains associated with *M. natalensis* belong to the same biological species [7,16,20,21] and because the shape of its nodules can be studied with the naked eye. A subset of 110 fungus combs from 12 colonies were carefully transferred to plastic zip-lock bags. The zip-lock bags were transferred to the laboratory in a plastic container and kept overnight at 4°C.

The next day, wet, sterilized chromatography or filter paper was placed inside a sterile Microbox container (model O118/50+OD118, white filter), and 2 ml of sterilized, demineralized water was added to each container to maintain high humidity. Fungus combs were transferred to each Microbox and any remaining termites were removed using sterilized forceps. The chambers were incubated in the dark at 25°C. The fungus combs were regularly inspected for mushroom formation (electronic supplementary material, table S1). In line with previous observations, as many as 29 combs were overgrown with other fungi, mainly *Pseudoxylaria*, within 4 days of incubation (electronic supplementary material, table S1) [22–24]. These 29 fungus combs were removed.

### (b) Basidiospore germination

To check basidiospore viability, spore prints were made from three mushrooms of different combs on agar plates. The cap of the mushroom was cut off and attached with Vaseline to the lid of a Petri dish with malt yeast extract agar (MYA) medium (20 g malt, 2 g yeast extract, 15 g agar in 1 l of demineralized water) for time periods ranging from 10 s to 1 h. After incubation at approximately 25°C, germinating spores were individually transferred to a fresh Petri dish with MYA medium. All mushrooms produced viable homokaryotic spores, which were confirmed by mating experiments.

### (c) Fixation and embedding of nodules

Normal nodules and primordia were carefully taken off from a fungus comb using a small brush. Thin slices of opposite vertical sides of the nodules were cut off to increase fixation speed and accessibility during infiltrations and allow positioning of the nodules in embedding moulds. Nodules were put in at least five times their volume of fixative (4% paraformaldehyde, 0.1% glutaraldehyde, and 0.05% Triton P40 in 0.05 M PBS pH 6.8) and submerged by creating a low pressure until they sunk. Samples were kept at 4°C until embedding.

Fixed samples were dehydrated for at least 10 min in 10%, 30%, 50%, 70%, 90% and two times in 100% ethanol followed by gradual resin infiltration (Technovit 7100 (T7100); resin A: 100 ml T7100, 1 bag of hardener I and 2.5 ml PEG 400). Samples were gently rotated for a minimum of 1 h at 30 rpm with resin A: ethanol mixtures (resp. 1 : 3, 1 : 1 and 3 : 1), followed by o/n rotation in 100% T7100 infiltration solution (A). Bottoms of the moulds were covered with a small layer of T7100 polymerization solution (resin B: 15 ml infiltration solution A and 1 ml hardener II). Samples were quickly transferred, oriented and covered with polymerization solution. Moulds were covered with a sheet of plastic, kept at RT for 1 h, followed by 37°C incubation for 1 h. Hardened embedded blocks were attached to microtome sample holders with freshly made Technovit 3040 glue. Longitudinal midplane sections (4 µm) were made, stretched on a water bath and baked to slides at 80°C.

### (d) Staining and imaging sections

Sections were stained for 15 s with Toluidine blue O (Merck 1.15930) (1% (w/v) Toluidine blue O in 1% potassium tetra borate, washed three times for 5 min in water and enclosed in Euparal permanent mounting agent. Sections were imaged in a Nikon 80i microscope with 20 × Plan Fluor 0.5 NA and 40 × Plan Fluor 0.75 NA objectives and a DS Fi1 colour camera. When needed images were stitched using Image Composite Editor (V2.0.3.0, Microsoft research).

## 3. Results

**Figure 1.**
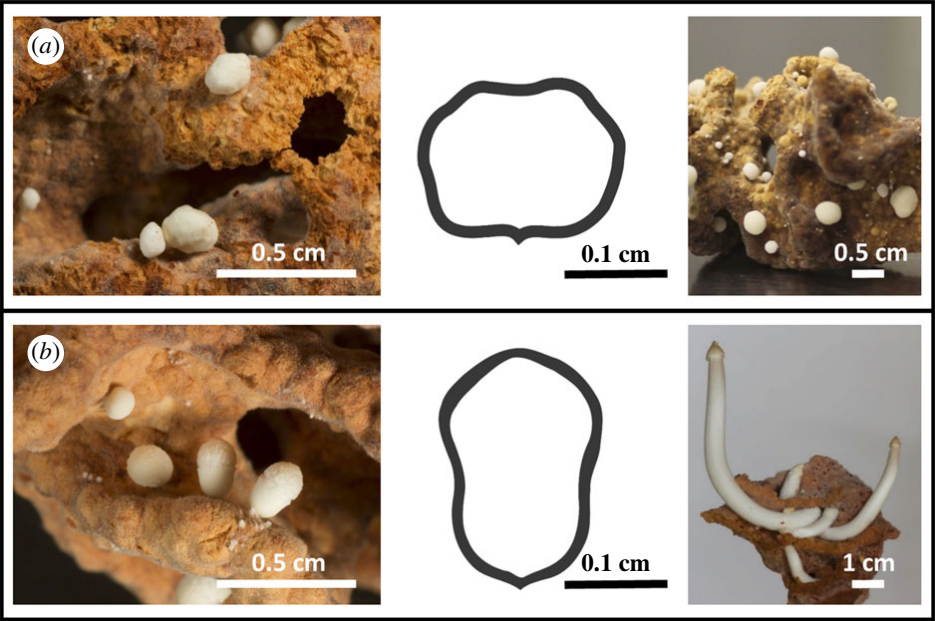
Two types of developmental structures found within mounds of *M. natalensis*: (*a*) normal nodules (left), fungus comb fragment, (middle) schematic drawing and (right) fungus comb fragment incubated without termites for 5 days showing enlarged normal nodules. (*b*) Primordia (left), fungus comb fragment, (middle) schematic drawing, (right) mushrooms growing from primordia after 4 days of incubation without termites. The front of the fungus comb has been broken off, to fully show the mushroom stipes. Cap of the mushroom already shows the typical *Termitomyces* perforatorium [10], i.e. the sharply pointed cap.

Unexpectedly, when we excavated the termite mounds, we observed that there were two different types of structures: the normally described, irregularly shaped roundish nodules as well as distinctly differently shaped structures that could, however, easily be mistaken for nodules (figure 1*a*). The shape of the latter was oval with a pointy top, and we hypothesized that these were true mushroom primordia (figure 1*b*). Over 3 years, we excavated 25 termite mounds, some of which in multiple years, adding up to 32 observations (electronic supplementary material, tables S1 and S2). We noted potential primordia in six different mounds at seven observations. On each comb fragment with potential primordia, less than 20% of all fungal developmental structures were regular nodules.

Of the 110 incubated fungus combs (electronic supplementary material, table S1), 91 only displayed normal nodules or no nodules and 19 displayed potential primordia. On average each comb contains more than 10 nodules, meaning that we studied over 1100 developmental structures, of which about 900 were nodules and about 200 were potential primordia. When incubated in the absence of termites, none of the normal nodules developed into mushrooms, whereas six combs with potential primordia developed fully grown, spore-producing mushrooms. On all combs with potential primordia, there were also potential primordia that did not develop into mushrooms. These primordia were arrested at different stages of development and some of them turned brown and wilted. One comb fragment in our experiment, in which all normal nodules turned brown and wilted—taken from a mound with only normal nodules—produced primordia after 16 days of incubation. These primordia also developed into mushrooms.

**Figure 2.**
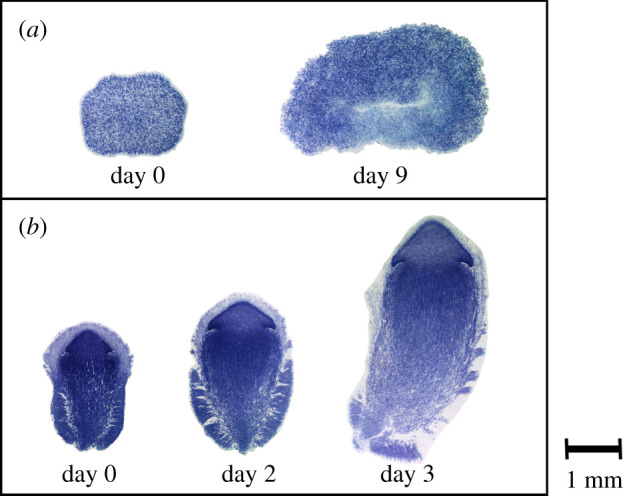
Images show toluidine blue stained midplane sections of different developmental stages of (*a*) nodules versus (*b*) primordia after incubation in the absence of termites.

The sections of potential primordia and their development showed that these developmental structures are indeed the true primordia of *Termitomyces* mushrooms (figure 2*b*; electronic supplementary material, figure S1A,B,C). By contrast, the sections of normal nodules did not show the hyphal alignment that is typical for mushroom formation (figure 2*a*), but rather showed unorganized strings of ovoid asexual spores and larger spherical cells (electronic supplementary material, figure S1E,F). Moreover, the larger nodules that were studied after 9 days did not develop mushroom features either.

## 4. Discussion

We tested the assumption that nodules are unripe mushrooms. We reject this assumption by showing that (i) normal nodules do not develop into mushrooms and (ii) although *Termitomyces* primordia bear resemblance to nodules, they are macro- and microscopically different developmental structures. Our observations of normal nodules confirm earlier descriptions of normal nodules in other species [9,10,25,26], and it is likely that our findings can be translated to all other *Termitomyces* species, as all known species make the nodules that are unique to this genus of fungi that are grown by termites [3,4].

Although our results showed that nodules are not the initials of mushrooms, this does not prove or disprove that fruiting body formation in *Termitomyces* is actively suppressed by its host. *Termitomyces* primordia may, similar to nodules, be consumed by termites, but this remains to be tested. Behavioural studies in these termites are, however, notoriously difficult, as termites immediately repair open areas in their mounds. Li *et al.* have recently managed to set up a laboratory colony of *Odontotermes formosanus*, which opens up possibilities for future studies, including behavioural ones [27].

Regardless of whether primordia are or are not consumed, the triggers for primordia formation are unknown. We only observed primordia in 20% of the excavations and when we observed primordia, an adjacent mound of the same species often did not have primordia. This indicates that there are factors within a colony that trigger or prevent primordia formation*.* We observed that combs that carried primordia were relatively mature in the sense that their colour was light, which is an indication of lignin breakdown and thus substrate depletion [28,29]. Also, we observed the formation of primordia on a fungus comb that had been incubated without termites for 16 days and was thus nutritionally depleted. Finally, it is known for other basidiomycete species mushrooms can be formed in response to starvation [30–32]. Therefore, we hypothesize that when fewer workers are present to maintain the fungus combs, some combs are left unattended and become nutritionally depleted because new substrate is no longer added. This nutritional depletion could, under the right environmental conditions, trigger the formation of primordia. Our hypothesis is in line with the observation that *Termitomyces microcarpus* mushrooms are found on pieces of comb that are ejected from a termite colony (thus left unattended) and with the observation that mushrooms are sometimes found on dead, unattended colonies [3,12,13].

Analogously, in the convergently evolved obligate ant–fungus symbiosis, the conflict over symbiont dispersal is mitigated by ant control over symbiont dispersal [33]. If the ant fungus is grown on substrate that is poor in protein mushroom formation is triggered. However, if the fungus is grown on substrate that is too rich in protein, vegetative growth is hampered. Mushroom formation in the ant fungus is suppressed by growing it on substrate that contains enough protein to prevent mushroom formation, but not so much that fungal growth is hindered [34].

## Supplementary Material

Cross section enlargements of pointy and normal nodules

## Supplementary Material

Fungus comb incubations in absence of termites and excavated colonies including their GPS coordinates

## Data accessibility

The datasets supporting this article have been uploaded as part of the electronic supplementary material.
