# Supplementary material for: Asexual and sexual reproduction are two separate developmental pathways in a Termitomyces species
Source: Biol Lett. 2020 Aug 12;16(8):20200394. doi: 10.1098/rsbl.2020.0394 (PMC7480157; doi:10.1098/rsbl.2020.0394)
Supplement: Cross section enlargements of pointy and normal nodules [file rsbl20200394supp1.docx]

Supplementary Figure to:

Asexual and sexual reproduction are two separate developmental pathways in a *Termitomyces* species

*Sabine M.E. Vreeburg^1^*, Norbert C.A. de Ruijter^2^, Bas J. Zwaan^1^, Rafael R. da Costa^3^, Michael Poulsen^3^, Duur K. Aanen^1^**

Published in Biology Letters

^1^Department of Plant Sciences, Laboratory of Genetics, Wageningen University, Wageningen, The Netherlands. ^2^Department of Plant Sciences, Laboratory of Cell Biology, Wageningen University, Wageningen, The Netherlands. ^3^Ecology and Evolution, University of Copenhagen, Copenhagen, Denmark.

*Correspondence: [sabine.vreeburg@wur.nl](mailto:sabine.vreeburg@wur.nl)


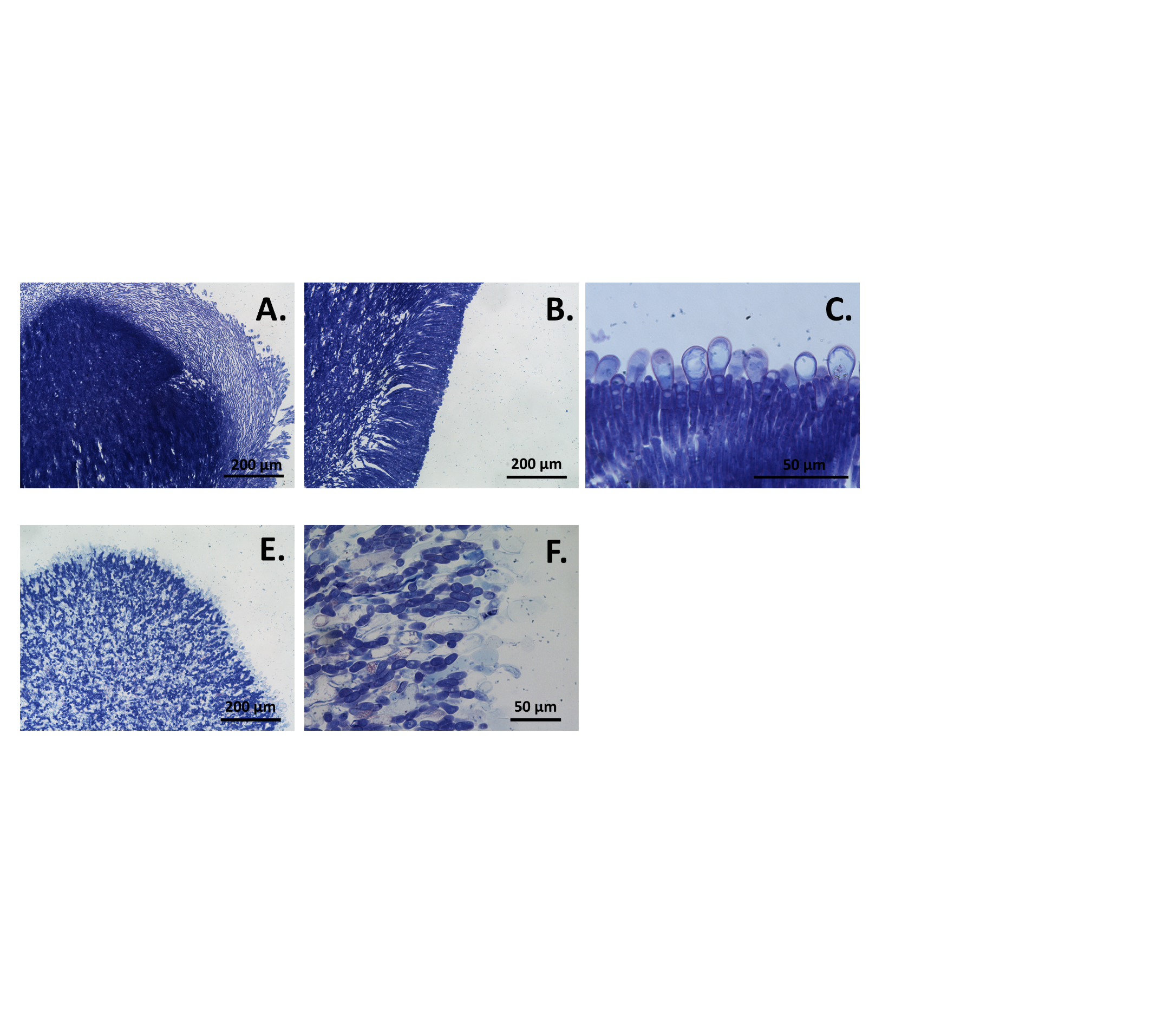


Supplementary Figure 1. Cross section enlargements of pointy nodules (A, B) and pointy nodule after 4 days of incubation without termites (C). (A) Pointy nodules show the start of cap formation and (B) parallel orientation of hyphae at the outside of the stipe. After 4 days of incubation without termites (B) lamellae were present, yet basidia were not mature enough to observe spore formation. Cheilostydia are clearly visible between basidia, which has been observed for many Termitomyces species [1-4]. Cross section enlargements of normal nodules (E, F). Normal nodules show unorganised aggregation (E) of hyphae, ovoid conidiospores and larger cells (F).

# References

1. Heim, R., *Termites et champignons: les champignons termitophiles d'Afrique noire et d'Asie méridionale*. 1977: Bouhée.

2. Wei, T.-Z., et al., *Termitomyces bulborhizus sp. nov. from China, with a key to allied species*. Vol. 108. 2005. 1458-62.

3. Pegler, D.N. and M. Vanhaecke, *Termitomyces of Southeast Asia.* Kew Bulletin, 1994. **49**(4): p. 717-736.

4. Van Der Westhuizen, G.C.A. and A. Eicker, *Species of Termitomyces occurring in South Africa.* Mycological Research, 1990. **94**(7): p. 923-937.
