# Supplementary material for: Asexual and sexual reproduction are two separate developmental pathways in a Termitomyces species
Source: Biol Lett. 2020 Aug 12;16(8):20200394. doi: 10.1098/rsbl.2020.0394 (PMC7480157; doi:10.1098/rsbl.2020.0394)
Supplement: Fungus comb incubations in absence of termites and excavated colonies including their GPS coordinates [file rsbl20200394supp2.docx]

Supplementary Tables to:

Asexual and sexual reproduction are two separate developmental pathways in a *Termitomyces* species

*Sabine M.E. Vreeburg^1^*, Norbert C.A. de Ruijter^2^, Bas J. Zwaan^1^, Rafael R. da Costa^3^, Michael Poulsen^3^, Duur K. Aanen^1^**

Published in Biology Letters

^1^Department of Plant Sciences, Laboratory of Genetics, Wageningen University, Wageningen, The Netherlands. ^2^Department of Plant Sciences, Laboratory of Cell Biology, Wageningen University, Wageningen, The Netherlands. ^3^Ecology and Evolution, University of Copenhagen, Copenhagen, Denmark.

*Correspondence: [sabine.vreeburg@wur.nl](mailto:sabine.vreeburg@wur.nl)

*Supplementary Table 1. Fungus comb incubations in absence of termites and mushroom development. Including observation remarks made during the experiment.*

| **Mound** | **Pointy nodules** | **nr. of incubated combs** | **nr. of combs with pointy nodules** | **Notes** |
| --- | --- | --- | --- | --- |
| Mn147 | No | 18 | 0 | day 4: on 8/18 nodules are browning, 5/18 no change, 5/18 contaminated day 12: all combs contaminated |
| Mn148 | No | 14 | 0 | day 4: 10/14 contaminated, 2/14 no change, 2/14 increasing nodule size day 12: 12/14 contaminated day 18: all combs contaminated |
| Mn132 | Yes | 12 | 9 | day 4: 9/12 different stages of mushroom development & normal nodules increasing in size 3/12 increasing nodule size day 6&7&9: spore prints made of fully developed mushrooms (from 3 different combs), 5/12 fully developed mushrooms, 4/12 different stages of mushroom development & normal nodules increasing in size, 3/12 very large, normal nodules day 12: all remaining combs contaminated |
| Mn161 | Yes | 13 | 10 | day 2: 10/13 pointy nodules increasing in size, 3/13 young comb, no nodules day 5: 3/10 pointy-nodule-combs no change, 3/10 pointy nodules browning, 3/10 used, 1/10 fully developed mushrooms, 2/3 no-nodule combs no change, 1/3 contamination |
| Mn162 | No | 2 | 0 | day 2: tiny nodules, very fresh comb |
| Mn149 | No | 14 | 0 | day 3: no obvious change day 11: all combs contaminated or dried out |
| Mn163 | No | 10 | 0 | day 2: 8/10 no obvious change, 2/10 nodules browning |
| Mn153 | No | 9 | 0 | day 7: 4/9 contamination, 5/9 no obvious change day 12: 5/9 contamination, 4/9 old nodules brown, new nodules growing on old nodules |
| Mn154 | No (Yes) | 8 | (1) | day 7: 7/8 contamination, 1/8 no obvious change day 12: 1/8 nodules start browning day 16: 1/8 old nodules brown, new pointy nodules appear on comb |
| Mn155 (dead) | No | 3 | 0 | day 4: 1/3 contamination, 2/3 large nodules day 9: 2/3 contamination, 1/3 large nodules day 12: 1/3 nodules start browning |
| Mn156 | No/No | 6 | 0 | only incubated fragments of first digging day day 4: 1/6 contamination, 5/6 no obvious change day 9: 5/6 contamination, 1/6 nodules start browning day 12: all combs contaminated |
| Mn160 | No | 1 | 0 | day 7: 1/1 huge nodules |

*Supplementary Table 2 Excavated termite mounds in January and February 2015, 2016 and 2018, including exact GPS locations and excavation dates. In total 25 mounds were dug of which five in multiple years, adding to 32 observations to show whether a mound contained pointy nodules (Yes), or only normal nodules (No). Mound Mn155 was dead at excavation, i.e., did not have a live queen and king, yet still contained uncontaminated fungus combs.*

| **Mound** | **Location** | **Pointy nodules** | | |
| --- | --- | --- | --- | --- |
|  |  | **2015** | **2016** | **2018** |
| Mn147 | S24 40.478 E28 47.898 | No | - | - |
| Mn148 | S24 40.509 E28 47.952 | No | - | - |
| Mn132 | S24 40.484 E28 48.271 | Yes | Yes | No |
| Mn161 | S24 39.668 E28 47.555 | Yes | - | - |
| Mn162 | S24 39.693 E28 47.559 | No | - | - |
| Mn165 | S24 39.724 E28 47.608 | - | No | - |
| Mn166 | S24 39.666 E28 47.590 | - | No | - |
| Mn173 | S24 39.694 E28 47.588 | - | Yes | - |
| Mn187 | S24 40.434 E28 48.275 | - | - | No |
| Mn188 | S24 40.512 E28 48.260 | - | - | Yes |
| Mn149 | S25 43.698 E28 14.102 | No | - | - |
| Mn163 | S25 43.761 E28 14.167 | No | - | - |
| Mn168 | S25 43.708 E28 14.461 | - | No | - |
| Mn169 | S25 43.666 E28 14.458 | - | No | - |
| Mn190 | S24 40.512 E28 48.260 | - | - | Yes |
| Mn153 | S25 44.492 E28 15.663 | No | No | - |
| Mn154 | S25 44.581 E28 15.659 | No (Yes) | No | No |
| Mn155 (dead) | S25 44.537 E28 15.659 | No | - | - |
| Mn156 | S25 44.623 E28 15.655 | No | - | No |
| Mn160 | S25 44.578 E28 15.645 | No | Yes | - |
| Mn164 | S25 44.762 E28 15.434 | - | No | - |
| Mn186 | S25 44.600 E28 15.648 | - | - | No |
| Mn171 | S25 56.622 E30 35.869 | - | No | - |
| 2004MN2.1ISWEPE | S26 48.898 E30 42.667 | - | No | - |
| DUURMN2004-3-2 | S26 50.163 E30 30.490 | - | No | - |
